# The financing gaps framework: using need, potential spending and expected spending to allocate development assistance for health

**DOI:** 10.1093/heapol/czx165

**Published:** 2018-02-05

**Authors:** Annie Haakenstad, Tara Templin, Stephen Lim, Jesse B Bump, Joseph Dieleman

**Affiliations:** 1 Harvard TH Chan School of Public Health, Department of Global Health and Population, 655 Huntington Avenue, Boston, MA 02115, USA; 2 Stanford University School of Medicine, Department of Health Research and Policy, 150 Governor's Lane, Stanford, CA 94305, USA and; 3 Institute for Health Metrics and Evaluation, University of Washington, 2301 5th Ave, Suite 600, Seattle, WA 98121, USA

**Keywords:** Overseas development assistance, aid, resource allocation

## Abstract

As growth in development assistance for health levels off, development assistance partners must make allocation decisions within tighter budget constraints. Furthermore, with the advent of comprehensive and comparable burden of disease and health financing estimates, empirical evidence can increasingly be used to direct funding to those most in need. In our ‘financing gaps framework’, we propose a new approach for harnessing information to make decisions about health aid. The framework was designed to be forward-looking, goal-oriented, versatile and customizable to a range of organizational contexts and health aims. Our framework brings together expected health spending, potential health spending and spending need, to orient financing decisions around international health targets. As an example of how the framework could be applied, we develop a case study, focused on global goals for child health. The case study harnesses data from the Global Burden of Disease 2013 Study, Financing Global Health 2015, the WHO Global Health Observatory and National Health Accounts. Funding flows are tied to progress toward the Sustainable Development Goal’s target for reductions in under-five mortality. The flexibility and comprehensiveness of our framework makes it adaptable for use by a diverse set of governments, donors, policymakers and other stakeholders. The framework can be adapted to short‐ or long‐run time frames, cross‐country or subnational scales, and to a number of specific health focus areas. Depending on donor preferences, the framework can be deployed to incentivize local investments in health, ensuring the long-term sustainability of health systems in low- and middle-income countries, while also furnishing international support for progress toward global health goals.

Key Messages

- Relying predominately on gross national income (GNI) per capita in aid allocation processes effectively penalizes economic growth and fails to capture contextual nuances important to channelling aid effectively and efficiently.
- We propose a ‘financing gaps framework’ that conceptualizes the allocation of development assistance for health based on empirical estimates of the gaps among three health financing trajectories: needed resources, expected spending and potential spending into 2030.
- Applying our framework to a case study of child health shows that priorities vary substantially when using our results as compared to GNI per capita or child mortality.
- The framework is flexible, adaptable to the wide-ranging priorities of development assistance partners and forward-looking, making it compatible with programmatic, planning and budgeting processes.

## Introduction

As growth in development assistance for health (DAH) levels off, development assistance partners must increasingly make allocation decisions within tighter budget constraints (IHME 2016; Dieleman *et al.* 2016). This entails difficult trade-offs among competing priorities and honing in on the specific challenges and countries most in need. Furthermore, the availability of comprehensive and comparable burden of disease and health financing estimates means new information can be incorporated into the allocation of DAH (Murray *et al.* 2015). Because of these factors, this is an apt moment to consider how new approaches could align funding flows with need.

Decision-making about aid flows is multifaceted, incorporating ethical, political, economic and programmatic considerations. Bilateral partners prioritize a diverse set of strategic, political and economic objectives (Meernik *et al.* 1998; Schraeder *et al.* 1998; Kuziemko and Werker 2006; Faye and Niehaus 2012). Economic performance, trade ties, good governance, media coverage and other factors have been associated with bilateral aid flows (Alesina and Dollar 2000; Berthélemy and Tichit 2004; Bandyopadhyay and Wall 2006; Eisensee and Stromberg 2007; Nunn and Qian 2014). Multilaterals consider many of the same components, including economic need, human development and good governance (Svensson 1999).

A number of multilaterals also deploy allocation formulas at the outset of aid decision-making processes (Maizels and Nissanke 1984; Frey and Schneider 1986). Most notable is the formula long-maintained by the World Bank’s International Development Association, which uses gross national income (GNI) as a key determinant of aid flows. Some members of the newest generation of multilaterals—notably the Global Fund to Fight AIDS, Tuberculosis and Malaria (the Global Fund) and Gavi, the Vaccine Alliance—also have explicit allocation formulas (Gavi, the Vaccine Alliance 2013; The Global Fund 2016a).

Chi and Bump (In press.) describe in detail the metrics used by health sector multilaterals in their allocation processes. The multilaterals surveyed are unified in employing measures of each organization’s health priorities to inform allocation decisions. However, GNI per capita generally forms the bedrock off which other considerations are developed. Other comparable and comprehensive empirical estimates contribute less often in a standardized, cross-cutting manner.

Using GNI as the core empirical input to funding decisions is problematic (Equitable Access Initiative 2016). First, basing aid allocation decisions on economic development penalizes countries for economic growth. In a GNI-based framework, less international assistance should be furnished as income grows. However, >70% of the world’s poor continue to reside in middle-income countries, and as income grows, the health needs of these populations persist (World Bank 2017). Second, the phase-out of aid could jeopardize progress in health. Health focus areas are diverse and countries may have shortfalls in specific focus areas irrespective of rising GNI. Finally, GNI per capita masks within-country inequalities and fails to capture contextual nuances critical to making aid effective.

We thus propose the ‘financing gaps framework’ as a practical, data-based tool which could serve as a starting point for allocating funds. The framework does not encapsulate the diversity of economic, political and ethical components considered, as we envision those elements coming into play farther along in the decision-making process. The flexibility of the framework makes it adaptable to a wide array development assistance partners and their health sector priorities. The parameters of the framework are meant to be adjusted for each organization, serving as one input into deliberations about the destination of health funding.

Our focus is on the gap between the resources needed to reach critical health targets and domestic health spending. We highlight two facets of domestic health resources—expected spending and potential spending—as critical. While donor preferences may vary, basing aid allocation on expected or existing spending levels incentivizes countries to spend less on health. We therefore propose the use of potential spending, which is a measure of a country’s ability to pay, as the domestic resource benchmark. Instead of the gap between expected spending and need, our framework focuses on the gap between potential spending and the health resources needed to meet global health targets. By focusing on that gap, donors can catalyse sustained domestic spending while also addressing the resource needs critical to reaching international health goals.

To demonstrate the potential of the financing gaps framework, we conduct a preliminary case study, applying the framework to the child health sector. This case study is merely an example of how the framework *could* be applied to one specific global health focus area and is not intended to be prescriptive or otherwise an authoritative statement on how donors should use their funds for child health. The case study demonstrates the feasibility of applying this framework and how aid might shift when the framework is deployed. We encourage donors and other decision-makers to assess the strength of this framework, as illustrated by the case study, and apply their own goals, timeline, models and assumptions to generate informed and empirical allocation decisions.

## Materials and methods

### Desirable features of a framework

To be practical and useful for donors, a number of key elements should be embedded in an allocation framework. First, we believe a tool should be grounded in empirical evidence. The availability of comprehensive and comparable health metrics means this is increasingly possible for a wide range of health focus areas. The scope of data availability also means a framework can be multidimensional, vital to representing a diversity of unique country contexts.

Second, we believe a framework should be goal-oriented. International goals have been important to focusing the attention of the international community over the last two decades and span many important areas of health. The Sustainable Development Goals (SDGs) are the newest in international targets (UNGA 2015), but global goals for HIV/AIDS, tobacco use and control, maternal health, child health and other health priority areas have also been articulated through other processes. With data disaggregated by geographic unit, wealth quintile or other measures of human development, goals can incorporate equity. In practical terms, it is now possible to integrate forward-looking, goal-oriented measures into allocation processes, as forecasts of health, health spending and the economy are recently available (Dieleman *et al.* 2017). Generally, the goal orientation of the framework is important to making aid an important catalyst for action.

Third, such a framework should be versatile and useable for a wide range of organizations, including national and local governments, donors, policy advocates or other global health stakeholders. The framework should be customizable to different contexts, health focus areas, preferences and needs. It should be easily understandable, so that governments and development assistance partners can explain and assess its utilization, ensuring accountability.

Fourth, it is critical that a framework not dis-incentivize domestic government or prepaid private health care spending. Incentivizing investments in health and the health system is key to long-term sustainability and avoiding aid dependency. Evidence suggests that aid is fungible, i.e. countries re-allocate aid away from the health sector as more DAH is disbursed (Pack and Pack 1990; Feyzioglu *et al.* 1997; Devarajan *et al.* 1999; Clements *et al.* 2004; Lu *et al.* 2010). This expenditure is not fully replaced when DAH retracts (Dieleman and Hanlon 2014). An allocation framework should be designed to prevent that kind of dynamic, without becoming a hard-and-fast condition. It should strike a balance between incentivizing investments in the health sector and not being overly prescriptive in how recipient countries spend government funds.

Our framework is designed to be a practical tool to help governments and donors identify the national and international spending needed to achieve global health goals. Both are important to the long-term impact of health sector investments and sustainability of health systems in low- and middle-income countries.

### Components of the financing gaps framework

**Figure 1:**
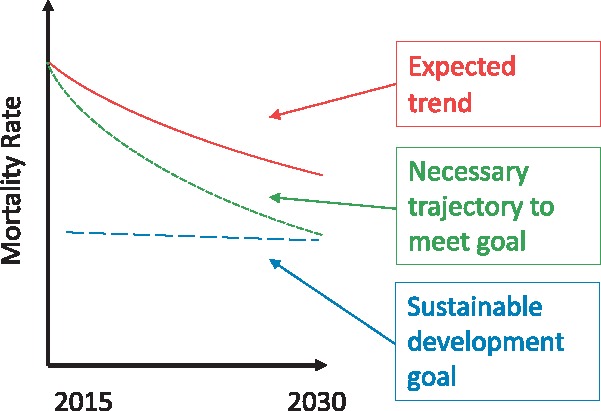
Representation of expected trend and trajectory necessary to meet international health goal

Figure 1 captures our conceptualization of a country’s trajectory toward a health target. In the example presented, the aim is to reduce an adverse health outcome, such as child mortality or HIV/AIDS incidence. The expected trend represents the course followed if current trends were extended over the period to the goal’s target date. The necessary trajectory captures the progress required to achieve the goal. In Figure 1, the SDG would not be met if the expected trend is not altered.

Of concern to development assistance partners is the gap between the expected trend and the trajectory necessary to meet a health goal. Altering the expected trend to match the health goal trajectory requires multifaceted approaches for catalysing progress. It also requires raising and disbursing funds to support those activities.

**Figure 2:**
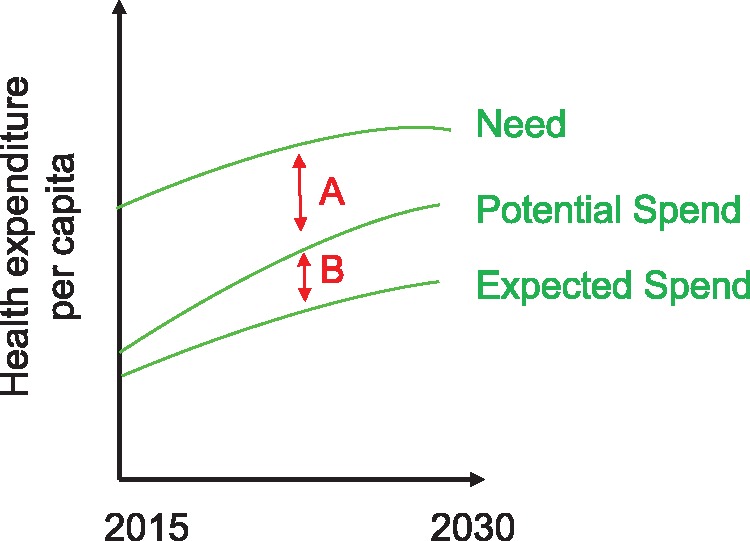
Comparing expected spend, potential spend and need

A critical component of our framework is associating the health goal trajectory with health spending. Need (N), as shown in Figure 2, represents the total resources required to reach a health goal—i.e. to put a country on the ‘necessary trajectory’ path. The cost of achieving the necessary trajectory, or closing the gap between expected trend and necessary trajectory, is represented by the gap between need and expected spend.

*Need* captures domestic governmental health spending, DAH, and potentially, prepaid private health expenditure. Expected spending (ES) is the resources contributed by governments or prepaid private sources, expected based on past trends and relationships. The contribution of prepaid private spending to need and expected spending may be of interest to certain international agencies, particularly in upper-middle income countries. Both measures encapsulate how far resources can go in a given country context, i.e. how efficiently investments can be deployed. Estimated costs, for example, are lower in countries with more capacity and greater efficiency, ceteris paribus.

Figure 2 highlights the other critical component of our framework: potential spending (PS). Potential spending is the governmental and prepaid private resources that could be disbursed to achieve a health goal. Potential spend is the maximum amount countries are expected to spend on health based on similar peer performers. Government spending on health per capita is driven by national income (Musgrove 1996) as well as a host of other factors including fiscal capacity. We therefore propose assessing potential spend of a given country according to the health spending of other countries in the same income group or similar along other dimensions important to raising funds. These include good governance, government capacity to enforce taxes or otherwise raise domestic revenues and other elements that may be important to donors. Potential spend is thus akin to a country’s ability to pay and is tailored to each country’s fiscal capacity, income and country context more generally.

The potential spend target is exogenous, and based on country peers, but is not intended to be a condition for receiving aid. The potential spending benchmark is also not designed to divert other social spending to the health sector. The measure is envisaged as a concrete target, aimed at focusing countries’ attention on health. It is also a benchmark against which donors can assess health spending. In our case study, potential spending is based on GDP per capita. However, the estimation of the potential spending could be as complex as a donor prefers, including custom capacity-to-spend analyses.

The gaps between the three lines identified in Figure 2 highlight the resources needed to meet the agreed upon prospective goal. For donors, assessing these gaps is critical to determining the allocation of scarce resources. More formally, the following equation identifies how our framework conceptualizes assessing the gaps to determine resource allocation:

DAH=α[(N−PS)+β(PS−ES)]

This conceptual framework highlights the two gaps illustrated in Figure 2. *N−PS* is Gap A. Gap A represents the difference between what a country could potentially spend and the need for health resources. Gap A is based on potential spend, which bifurcates expected spend and need. Gap A indicates the investments required to reach international health goals outside of a given country’s contribution.

*PS−ES* is Gap B. The divergence between expected spend and potential spend—represented by Gap B (PS*−*ES)—captures the potential for additional domestic spending. Gap B provides an indication of the country-level commitment to a given health goal and whether more funding could be mobilized domestically. We consider this gap to be multidimensional, capturing a number of features of country decision-making, not limited to willingness-to-pay and the prioritization of other areas of social investment. In certain circumstances, public financial management challenges could be reflected in Gap B—countries may have the will to invest in the sector but not the know-how.

In our framework, Gap B is valuable for incentivizing local spending and avoiding crowd-out of local investments, vital to the long-term sustainability of health systems. The sum of Gap A and Gap B is the total financing gap preventing the realization of health goals.

*α* and *β* represent weights to be determined by users of the framework. These weights represent scalars that must be selected to reflect the amount of resources available from the donor (*α*) and the importance the donor places on the framework incentivizing domestic health care spending or at least not dis-incentivizing domestic health care spending (*β*). In most cases, we expect *α* to be quite small, but greater than zero, as it reflects the share of the total financing gap (across all countries or subnational units being considered) that will be funded by the donor. Alternatively, we would expect that *β* be any value less than or equal to zero but could be as high as one depending on donor preferences.

In a scenario where a donor was not concerned with incentivizing additional domestic spending and is content dis-incentivizing domestic spending (i.e. *β* = 1), the equation would reduce to *α[N−ES]*. In this case, donors would focus on the financing gap between expected spending and needed resources only. Here the only constraint preventing donors from filling the financing gap is *α*. Funding based on the total financing gap (Gap A + Gap B) removes the incentive for domestic governments to fund their own health system, as it penalizes increases in domestic spending with the provision of less DAH.

If a donor aims to deploy the framework in a manner that does not incentivize or dis-incentivize domestic health spending, we would expect *α* > 0 and *β* = 0, however. In this scenario, the funding rule reduces to *α[N−PS]* and funding is independent of expected spending (ES) and focused exclusively on financing the Gap A between need (N) and potential spend (PS). Finally, if a donor aims to incentivize health spending, *β* can be set to less than zero. Setting *β* > 0 could incentivize poor performers and thus we anticipate aid agencies would not be inclined to use this value of the parameter.

Critical to the flexibility of this framework is *α* and *β*, which are designated by the donor institution. We leave it to funders to determine the magnitude of *α* and *β*, as donors should decide on the importance of each financing gap to their mission.

Estimation of each piece of our framework requires using observed trajectories to forecast future trends and outcomes. These forecasts, be it of health outcomes, resources needed or resources available, are vital for realization of the forward-looking nature of our framework. Despite the challenges associated with forecasting, some baseline set of expectations is essential for informed policy-making. These forecasts, of course, can vary in sophistication and rigor but should include as much empirical data as possible and, when feasible, should be empirically validated using out-of-sample validation techniques.

### Applying the resource allocation framework: a child health case study

To provide potential users with a sense of how the framework could be applied, we developed a case study focused on child health. This is not intended to indicate how DAH should be allocated for child health, but rather serve to demonstrate the feasibility of the financing gaps framework.

Quantifying the resources needed to meet an objective health goal is the first component. In general, the measurement of this component is likely to be based on a combination of population health data, marginal cost data associated with improving health outcomes and a health goal. Population health data can be obtained from an array of sources. In our case study, we rely on the Global Burden of Disease 2013 (GBD 2013), which estimates age-, sex- and cause-specific measures of prevalence, incidence, mortality, morbidity and disability-adjusted life years for each country in the world. More specifically, our case study makes use of GBD 2013 estimates of under-five mortality (5q0) and total, country-level population. The methods used to produce these data have been detailed at length (GBD 2015 Child Mortality Collaborators 2016). These data are publicly available online through visualizations (IHME 2017) and for public download. These data are particularly valuable for this exercise because of their comparable and comprehensive nature and considerable time span. In addition to national-level estimates, some subnational estimates may also prove useful for within country allocation purposes. These can be used to ascertain inequities in domestic disbursements, important for targeting need in large countries such as China and India.

Population health data need to be matched with international health goals. We used the target for child health under SDG 3. The target is to reduce under-five mortality to 25 per 1000 live births by 2030 (UNGA 2015). Many other health goals, such as 90–90–90 target for HIV/AIDS or those articulated in the Global Strategy for Women’s, Children’s and Adolescents’ Health could fit well in the framework as well (UNAIDS 2014; Every Woman Every Child 2015).

To estimate the resources needed to reach the stated health goals, historical health spending data, combined with trends in key health outcomes, should be used. Spending data should capture the cost per unit of the health measure targeted by the international goal. Through credible resource tracking exercises such as System of Health Accounts (SHA), National AIDS Spending Assessments, disease expenditure research and costing studies, valuable cause-of-illness financial information is ever improving and more readily available.

For our case study, spending estimates for child lives saved were extracted from recent work completed by Murray and Chambers (2015). Murray and Chambers (2015) estimate that the cost per child life saved is $4205 in low-income countries, $6496 in lower-middle income countries and $10 016 in upper middle-income countries. These estimates build off data from the World Health Organization, Institute for Health Metrics and Evaluation and National Health sub-accounts. Using the health intervention driven component of under-five mortality to develop counterfactual scenarios (Wang *et al.* 2014), Murray and Chambers compare ratios of changes in expenditures with changes in under-five deaths to estimate the incremental cost per life saved. These estimates take into consideration complex contextual factors such as health system capacity and efficiency. Cost estimates like those developed by Murray and Chambers, which incorporate those factors, are fundamental to the accuracy and relevance of this framework.

For the child health case study, we estimate the expected trend for mortality rates based on regression analysis. We regress mortality rates (natural log transformed) on gross domestic product (GDP), gross domestic product per capita, maternal education, DAH per capita, government health expenditure as source per capita and technology (proxied by a linear time trend). Maternal education and GDP were sourced from the GBD 2015 study (GBD 2016) and DAH and government health expenditure estimates were extracted from Financing Global Health 2015 (IHME 2016), both of which are publicly available. To generate the estimated baseline trends, we use this fitted model to predict future mortality rates based on forecasted GDP per capita, education rates and the changes in technology, and no change in donor or government health spending.

The resources needed to reach the SDGs are found by calculating the annual percent reductions in mortality required to reach the target. We multiply these required reductions by population estimates and take the difference between the expected trend and trajectory necessary to reach the SDG. We then extract the previously estimated country‐specific estimates of the cost to save a child life from Murray and Chambers (2015). Murray and Chambers use data drawn from National Health Account (NHA) child health sub-accounts, which capture spending on services and activities delivered to the child or its caretaker after the birth of the child and whose primary purpose is to restore, improve and maintain the health of children under 5 years of age (WHO 2012). Multiplying the cost to save a life by the number of additional lives that need to be saved generates our estimates of the resources necessary to meet the SDG target for child health.

### Expected spend

The second principle measurement is government resources expected to be spent to achieve health goals. To estimate expected spending, we utilized government health expenditure as a source (GHE-S), as estimated annually and provided in the Financing Global Health 2015 report (IHME 2016; Dieleman *et al.* 2016). This metric is generated by subtracting estimates of DAH disbursed to governments from World Health Organization’s Global Health Observatory data on general government health expenditure (WHO 2015). Out-of-pocket spending is not captured.

Child health subnational health accounts were used to quantify the expected spend specific for the case study. For countries with subnational health accounts, the fraction of total government spending (GHE-A) allocated to child health was regressed on log GHE-S per capita and log GDP per capita. Total government health spending as source (GHE-S) is predicted for each country in the world through 2040 as part of the Institute for Health Metrics and Evaluation’s broader forecasting project. While these data are sufficient for this case study as made possible by the major investments in the SHA 2011, more data are needed to track health spending by priority area across countries in a comparable manner. Government health spending is forecasted into the future using an ensemble model that averages across the broadest feasible set of models. The estimated coefficients from these models were used to forecast government health spending on child health through 2030.

### Potential spend

The third principle measurement is potential government health spending. Data envelopment analysis (DEA) was used to estimate a production frontier of potential government health expenditure, as a function of that country’s level of development as measured by GDP per capita (Charnes *et al.* 1978). Based on GDP per capita forecasts, potential spending estimates are forecasted through 2030 to estimate potential spend for each country and year. We follow the formulation of DEA presented by Di Giorgio *et al.* (2016) and implement the analysis using the package developed by Ji and Lee (2010).

The technical annex provides more details on the methods used to estimate need, expected spend and potential spend for the child health case study.

## Results

### Child health case study results

**Figure 3:**
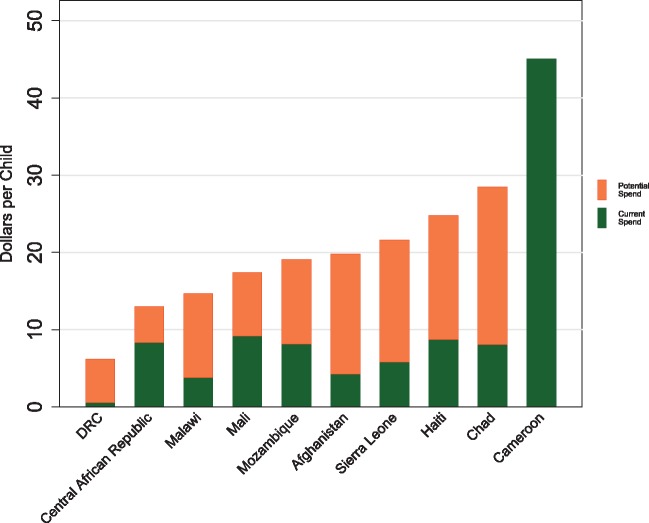
Potential and expected spending on child health for the 10 countries with the most need

The child health case study produced diverse results across countries. Figure 3 presents potential and current spending on child health for the 10 countries with the most need. These depict the gaps between potential spend and expected spend as well as the lack of adequate child health financing in some countries. Among the countries with most need for additional child health resources, the gap between expected spend and potential spend was highest in Afghanistan, at 79%, and lowest in Cameroon, where expected spend exceeded potential spend.

**Figure 4:**
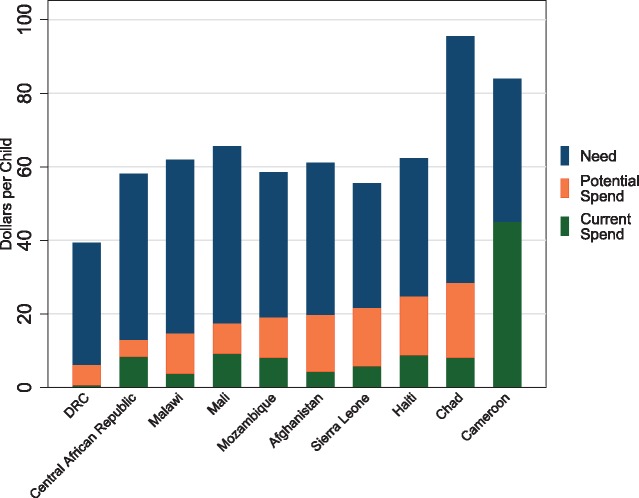
Resources needed and potential and expected spending on child health for the 10 countries with the most need

Figure 4 captures the gap between need and potential spend for child health. Large gaps between need and potential spend were present across the 10 countries with the most need in child health. They ranged from 84% in the Democratic Republic of the Congo to 46% in Cameroon.

**Figure 5:**
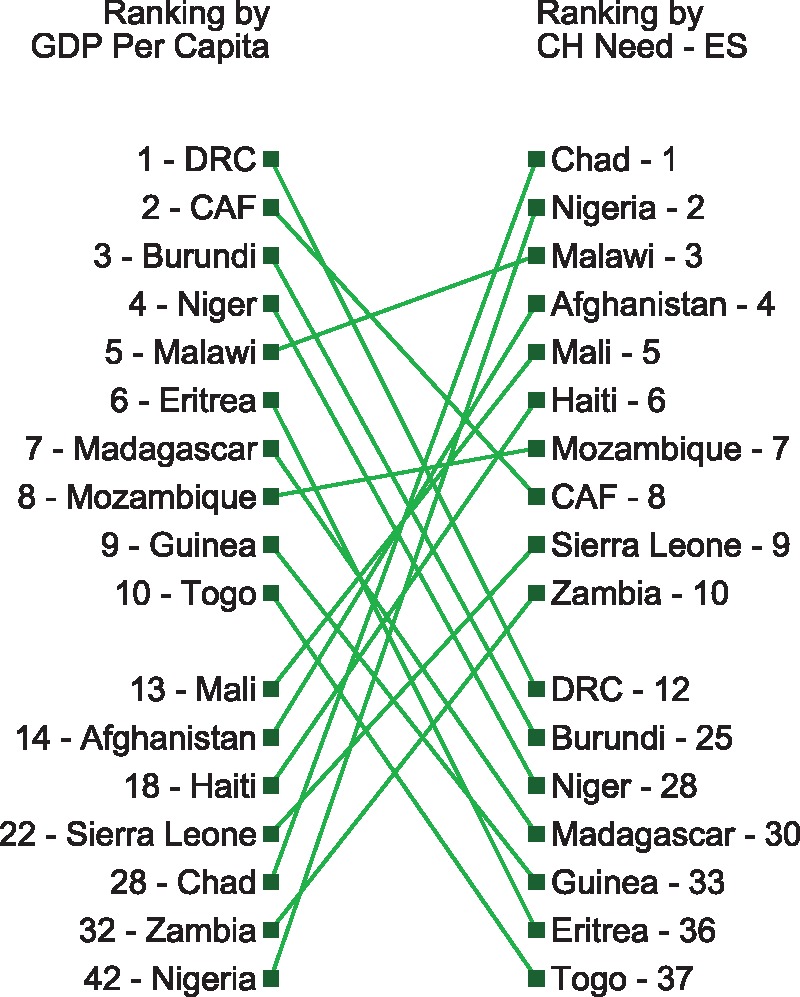
GDP per capita vs the gap between resources needed and expected government spending on child health (Gap A + Gap B)

**Figure 6:**
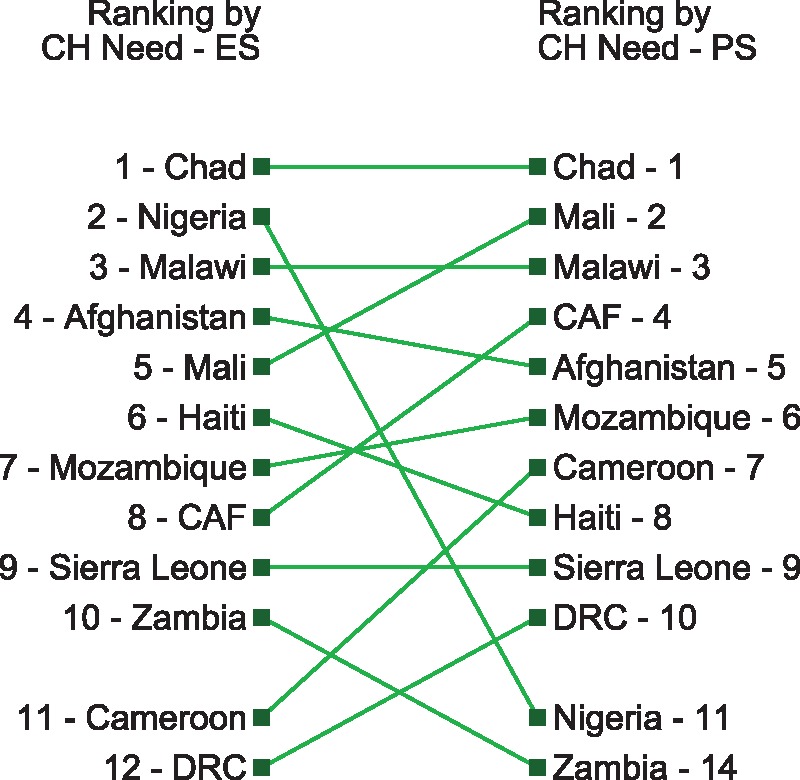
Gap between need and expected government spending on child health (Gap A + Gap B) vs the gap between need and potential government spending on child health (Gap A)

**Figure 7:**
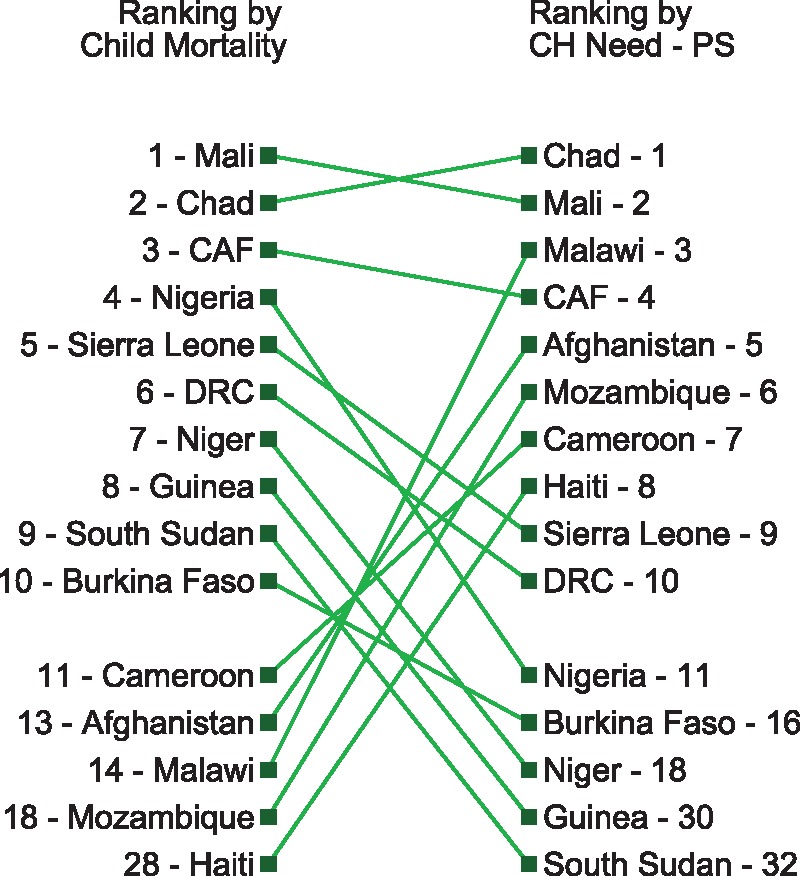
Unadjusted child mortality vs the gap between need and potential government spending on child health (Gap A)

In Figures 5–7, we rank each country based on different allocation strategies, highlighting how prioritization of countries would vary according to different donor preferences. While it is unlikely a development partner would allocate funds in a cascade down these lists, the ordering of countries with the greatest need would likely provide context for allocation and eligibility decisions. These figures underscore the importance of basing allocation and eligibility decisions upon criteria that is specific to the interest of the donor.

In Figure 5, the ordering of countries based on GDP per capita (on the left), a proxy for level of development, is contrasted with the ranking produced through the child health case study (on the right). The countries on the right are ordered depending on the size of the gap between need and expected spend (Gap A + Gap B). The column on the left deprioritizes countries such as Chad or Nigeria, which have more substantial GDP per person but high child mortality. Figure 5 shows that, in a strictly GDP per capita scenario, critical financing pitfalls—as captured by the gap between need and expected spending—may be missed.

Figure 6 highlights how priorities shift when the model relies on the difference between potential health spending and need (Gap A) rather than the gap between expected health spending and need (Gap A + Gap B). For example, using potential spend (Gap A), Mali is ranked second, whereas using expected spending (Gap A + Gap B) puts Mali at rank 5. Other countries spend less domestically, increasing their rank order on the list on the left. Basing aid allocation on expected spending deprioritizes Mali, penalizing existing government spending on child health. Spending less on child health would result in moving up the order of countries on the left.

Finally, Figure 7 depicts the differences in country ordering with child mortality as compared with the gap between need and potential spend (Gap A). With the exception of the first three countries (Chad, Mali and the Central African Republic), this figure displays major differences in the ranking produced through the two methods. Integrating ability to pay, represented by potential spending, emphasizes the need present in Haiti, Mozambique and Malawi.

## Discussion

Applying the financing gaps framework to the child health case study highlighted a number of its attributes. First, comparing rankings based on the child health with those based on economic development or child mortality showed how the suggested framework is likely to provide distinct information for allocation decisions. Because our framework revolves around a prospective goal, expected trends and country-specific costing data, our framework provides a valuable, empirical assessment.

Many health sector aid agencies have a mandate specific to a given health area. The financing gaps framework can bolster how progress particular to that health focus area is incorporated into allocation processes. For other organizations with a broader range of emphasis, the framework may be useful once allocation across areas has been decided. A multifaceted, cross-cutting version of the framework could be used to inform allocation across health focus areas.

Second, the priorities suggested by the child health case study change when using expected vs potential spending for quantification of the financing gap. The countries with more health spending dropped down the priority list when expected spending was used. This is largely counter to the objectives of development assistance partners. Distinguishing among resources needed, expected government resources available and potential government resources together provide incentives for both donors and governments.

Measuring the gaps separately allows users of the framework to decide for themselves how to respond to each of these distinct gaps. Gaps between expected and potential spend highlight tangible goals for government spending and potentially domestic prepaid private health expenditure. Aid agencies may want to incentivize a country to cover some portion of their nation’s health care, by focusing on financing the gap between need and potential spending (Gap A). By considering this gap only, donors can incentivize governments to progressively realize their health spending potential. There is already evidence of some deployment of this type of strategy. The Global Fund, for instance, incorporates counterpart financing into its funding model (The Global Fund 2016b). Conversely, organizations interested in filling wider financing gaps to meet health goals could focus on the entire space between resources needed and resources currently available (Gap A + Gap B).

Finally, because users of the framework define their own goals, there is flexibility to focus on different components of population health as well as objectives in other development sectors. UNAIDS, for example, compares forecasts of the expected HIV cases by 2030 with trajectories in line with 90–90–90 targets (UNAIDS 2016). Well-defined SDG targets for education and environment, for example, could also be used if good estimates of costs were also available. Short- or long‐term goals can be used to reflect organization-specific planning horizons and broader institutional goals. To extend the framework more generally to official development assistance would be challenging because there is no clear measure to represent all of these investments or improvements. However, composite indicators such as the human development index or multi-dimensional poverty index could potentially be used, if good estimates of the costs of progressing along several dimensions simultaneously could be developed.

While this framework has considerable potential, we recognize its limitations. The amount of data needed to execute this framework is substantial, and data may not be available for every health focus area. Currently, data on government health expenditure, and particularly government health expenditure on specific health focus areas, is of variable quality. This is a core data limitation of the child health case study. The availability and quality of cause-specific and costing data are improving, but still needs substantial improvements to be considered comprehensive. Furthermore, marginal cost data are not widely available and would need to be developed for many health focus areas. We know less about how marginal costs change as coverage is scaled, and this may limit applicability and usability for some donors. For anything beyond the most basic estimation, some modelling and assumptions will be needed. However, as long as development assistance partners are aware of these limitations, and how assumptions affect priority-setting, we believe that the financing gaps framework is a very useful tool for conceptualizing the allocation of DAH.

The other main limitation of this work pertains to the measurement and interpretation of potential spending. DEA analysis, which was used in our child health care case study, fundamentally rests on comparing countries based on unexplained variation—i.e. the residual. Omitted variables or measurement error can contribute to the size of the residual and the final ranking of countries in DEA. Potential spend estimates are based, generally, on this unexplained variation and thus should not be interpreted as tied directly to efficiency or any other single factor. Finally, we recognize that many factors important to the effectiveness of aid are not included in our framework, including the conditionalities associated with disbursements and whether aid is delivered vertically or in a way that strengthens the health system. The framework should thus be applied consistent with other important tenets of aid policy.

## Conclusions

Prioritizing and allocating resources across health focus areas and countries are complex. Fifty years ago, GNI was the best proxy for countries’ ability to finance their own development and health. Today, empirical estimates of wide range of health outcomes and health financing dimensions are available. A number of development assistance partners have taken advantage of this new era of information, integrating broader health and focus-specific metrics into their allocation processes. However, as Chi and Bump (In press) illuminate, development assistance partners continue to rely heavily on GNI per capita.

Our framework emphasizes how development assistance partners can further incorporate a broader set of health and health financing measures into their decision-making processes. Using empirical data and targets is an objective starting point for making decisions about the allocation of DAH. Explicitly considering targeted health measures can help donors focus on the issues most pertinent to their mandate, mobilize the domestic financing vital to sustainable health systems and ensure each dollar ultimately reaches those most in need across populations in the developing world.

## Funding

This work was supported by the Wellcome Trust [099114/Z/12/Z].

## Ethical approval

We rely on existing, non-Human Subjects data for this research and thus no ethical approval was required.

*Conflict of interest statement*. None declared.
